# Collaboratively building teaching resources for EDI in epidemiology and biostatistics

**DOI:** 10.17269/s41997-026-01225-7

**Published:** 2026-08-03

**Authors:** Japteg Singh, Todd Coleman, Agatha Nyambi, Tyler Pittman, Amanda Doggett, Fernanda Andre, Josée Dupuis, Daniel Fuller

**Affiliations:** 1 Department of Epidemiology, Biostatistics, and Occupational Health, McGill University, Montreal, QC Canada; 2 Department of Health Sciences, Wilfrid Laurier University, Waterloo, ON Canada; 3 Department of Psychiatry and Behavioural Neurosciences, McMaster University, Hamilton, ON Canada; 4 Department of Community Health and Epidemiology, University of Saskatchewan, Saskatoon, SK Canada; 5 Department of Heallth Research Methods, Evidence and Impact, McMaster University, Hamillton, Canada

**Keywords:** Equity, diversity and inclusion, Epidemiology and biostatistics, Gender-diverse, Race, Reflexivity, Teaching, Équité, Diversité et inclusion, Épidémiologie et biostatistique, Diversité de genre, Race, Réflexivité, Enseignement

## Abstract

**Objectives:**

The objective of this workshop was to collaboratively co-create concrete, practical teaching resources that embed equity, diversity, and inclusion (EDI) concepts into epidemiology and biostatistics teaching in Canada.

**Methods:**

The 3-h interactive session developed four examples of the inclusion of EDI considerations in epidemiology and biostatistics teaching based on commonly used textbooks. Participants, in small breakout groups, critically reviewed examples from two common textbooks (*Modern Epidemiology *and* Fundamentals of Biostatistics*). Groups of four participants selected two examples each from a list of core concepts (e.g., confounding, regression). The groups then integrated EDI considerations into the core examples from the textbooks by addressing focus areas like sex and gender or race and ethnicity, and presented their revised materials.

**Results:**

Two groups of four participants selected two topics each. Group 1 selected regression and missing data, while group 2 selected interaction/effect measure modification and hypothesis testing and two-way ANOVA. For a regression model example from *Modern Epidemiology*, participants recommended changing “pregnant women” to “pregnant person” for gender inclusivity and reframed “normal” to highlight social and structural determinants. In a missing data example, they replaced ageist and judgment-laden terminology (“elderly,” “unwilling”) with “older adults” and “unable.” Group 2 discussed the importance of including biological plausibility and measurement considerations when using sex, defined as male/female, as an effect measure modification in potential analyses.

**Conclusion:**

This workshop and concomitant paper demonstrate the need for integrating EDI into epidemiology and biostatistics teaching. It also provides concrete examples of how this can be done using common textbooks. Integrating EDI into the curriculum not only improves student understanding of EDI, but can also strengthen understanding of, and interest in, epidemiology and biostatistics.

## Résumé

**Objectifs:**

L’objectif de cet atelier était de cocréer de manière collaborative des ressources pédagogiques concrètes et pratiques intégrant des concepts d’équité, de diversité et d’inclusion (EDI) au sien de l’enseignement de l’épidémiologie et de la biostatistique au Canada.

**Méthodes:**

La séance interactive de 3 heures a permis d’élaborer quatre exemples d’intégration de considérations d’EDI dans l’enseignement de l’épidémiologie et de la biostatistique, à partir de manuels scolaires couramment utilisés. Les participants, répartis en petits groupes de discussion, ont examiné de manière critique des exemples tirés de deux manuels importants « *Modern Epidemiology*» et « *Fundamentals of Biostatistics*». Des groupes ont sélectionné deux exemples à partir d’une liste de concepts fondamentaux (p. ex., facteur de confusion, régression). Les groupes ont intégré des considérations d’EDI aux exemples issus des manuels scolaires, en abordant des thèmes tels que le sexe et le genre ou la race et l’ethnicité. Chaque groupe a présenté ses exemples révisés.

**Résultats:**

Deux groupes de quatre participants ont chacun sélectionné deux thèmes. Le premier groupe a choisi la régression et les données manquantes, tandis que le deuxième groupe a sélectionné l’interaction/la modification de la mesure d’effet ainsi que les tests d’hypothèse et l’ANOVA à deux facteurs. Dans le premier groupe, pour l’exemple de modèle de régression tiré de « *Modern Epidemiology*», les participants ont recommandé de remplacer « femmes enceintes» par « personnes enceintes» afin d’assurer une plus grande inclusivité du genre, et ont reformulé le terme « normal» afin de mettre en évidence les déterminants sociaux et structurels. Dans un exemple sur les données manquantes, ils ont remplacé une terminologie âgiste et porteuse de jugement « personnes âgées», « réticent» par des expressions plus appropriées « adultes plus âgés», « incapables». Le groupe 2 a discuté de l’importance d’inclure la plausibilité biologique et les considérations liées à la mesure lorsque le sexe, défini comme masculin/féminin, est utilisé comme modificateur de la mesure d’effet dans des analyses.

**Conclusion:**

Cet atelier et l’article qui l’accompagne démontrent la nécessité d’intégrer l’EDI dans l’enseignement de l’épidémiologie et de la biostatistique. Ils fournissent également des exemples concrets de la manière dont cela peut être réalisé à partir de manuels scolaires courants. L’intégration de l’EDI dans le programme améliore non seulement la compréhension des étudiants en matière d’EDI, mais peut également renforcer leur compréhension de concepts clés en épidémiologie et en biostatistique, ainsi que leur intérêt pour ces disciplines.

## Introduction

Epidemiology and biostatistics are often taught through problem-based learning. However, example-based problem sets are frequently oversimplified and may not fully incorporate principles of equity, diversity, and inclusion (EDI). For instance, using only two gender categories in a problem set on binary data may inadvertently reinforce restrictive assumptions about gender. Incorporating more inclusive examples can prompt students to critically consider how identity and context shape data and analysis. Such omissions influence how future epidemiologists and biostatisticians conceptualize populations, frame research questions, and interpret findings. This exclusion is increasingly recognized as a limitation to both the generalizability and social relevance of epidemiologic research (Banerjee et al., 2023; Braveman, 2001). In recent years, efforts to integrate EDI within epidemiology education have expanded across Canada, yet challenges remain in translating these commitments into tangible teaching practices (Goldmann et al., 2018).

At the 2025 Canadian Society for Epidemiology and Biostatistics (CSEB) conference in Montréal, a workshop was held to advance efforts to integrate EDI into epidemiology and biostatistics education. This session built on the successful 2023 CSEB workshop, “Incorporating EDI into the Epidemiology and Biostatistics Curriculum and Classroom,” which introduced nine foundational strategies for inclusive teaching (Fuller et al., 2024). The 2025 workshop focused on moving from theory to practice through the development of practical teaching materials. Through facilitated breakout groups, participants critically reviewed common teaching examples from *Modern Epidemiology *(Rothman et al., 2008) and *Fundamentals of Biostatistics *(Rosner, 2006) and re-envisioned them to better reflect the diversity of learners, populations, and health experiences in Canada. This collaborative process provided a platform to examine how equity considerations can be systematically integrated into methodological instruction, advancing the practical implementation of EDI in teaching and curriculum design.

This paper summarizes the key activities and outputs of the 2025 workshop and reflects on lessons learned for integrating EDI principles into epidemiology and biostatistics education. By documenting these collaborative approaches and highlighting the worked examples developed during the session, we aim to inform ongoing curriculum development and support educators seeking to foster more inclusive and contextually relevant training environments.

## Methods

This workshop was designed to provide a structured yet interactive environment for participants to explore strategies for embedding EDI principles into epidemiology and biostatistics instruction.

### Goals and learning outcomes

The workshop aimed to achieve four objectives: (1) discuss the importance of integrating EDI principles into epidemiology and biostatistics teaching; (2) assess and share current strategies used to incorporate EDI in existing curricula; (3) develop new teaching resources and case examples that reflect inclusive frameworks and real-world complexities; and (4) identify barriers and facilitators to embedding EDI principles into teaching, including the influence of institutional and political contexts.

The intended learning outcomes were that participants would (a) critically assess current course materials through an EDI lens, (b) gain practical tools for revising examples and exercises, and (c) strengthen their ability to engage students in discussions about social identity, power, and structural determinants of health.

### Recruitment and participation

All workshop participants were invited, and agreed, to be authors on the present manuscript. Workshop participants were recruited through the Canadian Society for Epidemiology and Biostatistics conference registration process and related conference communications. The workshop was offered as an interactive, small-group session to allow for discussion, collaborative revision of teaching materials, and facilitated reflection. Registration was open to conference attendees and was not capped; the workshop did not reach capacity. Eight participants attended the workshop, including three faculty members, four PhD students, and one postdoctoral fellow, all working in health research or biostatistics. We did not collect individual-level demographic information from participants. One participant had attended the previous workshop in 2023. Participants were informed that workshop discussions and group outputs, including revised teaching examples and emerging themes, would be summarized to inform a manuscript. No individual participants are identified in the manuscript.

### Structure and facilitation

The 3-h interactive workshop was facilitated by five educators and researchers with expertise in epidemiology, health equity, and inclusive pedagogy. Each facilitator brought a distinct perspective grounded in reflexivity and lived experience. Positionality statements were shared at the outset to model transparency and reflexivity in teaching. The facilitation team was made up of faculty and trainees working in the field of epidemiology and/or biostatistics, and included individuals from diverse social backgrounds, encompassing experiences in immigrant health, 2SLGBTQIA + health equity, and Indigenous data governance.

A structured format was followed, beginning with an introduction to foundational EDI concepts and a discussion of how traditional approaches to epidemiologic teaching have often lacked attention to inclusion and representation. Participants were then guided through a land acknowledgement recognizing the unceded territory of the Kanien’kehá:ka Nation, emphasizing that reconciliation requires equity, diversity, and inclusion efforts in Canada to be informed by and grounded in Indigenous knowledge systems. Ground rules were established to foster openness, active listening, and respect for difference. These included:

1. Be proactive: contribute actively to discussions and group work;
2. Listen actively: engage with others’ perspectives with curiosity and openness;
3. Speak from experience and relate insights to public research;
4. Be respectful;
5. Remember the goal is to learn.

Participants were divided into two small breakout groups of four. Each group selected two core epidemiology or biostatistics teaching concepts from the list provided by the facilitation team, which included: incidence/prevalence, regression, and confounding. interactions/effect measure modification, selection bias, measurement error, weights/standardization, population inference/target population, directed acyclic graphs (DAGs)/causal inference. After choosing their topics, groups were given corresponding examples from *Modern Epidemiology* or *Fundamentals of Biostatistics* and tasked with integrating EDI considerations into these materials. Since topics were self-selected by participants from the list provided, the final set of revised examples reflected participant interests and included three biostatistics-focused examples and one epidemiology-focused example. The facilitation team supplied printed textbook excerpts for each concept to guide this process. Groups then selected an intersecting EDI focus area such as sex and gender, race and ethnicity, ageism, or disability, and worked collaboratively to revise the material or suggest alternative EDI-focused examples.

Each group was allotted 90 min to revise selected textbook materials to incorporate EDI considerations. Facilitators circulated among the groups to provide guidance and prompt reflection on positionality, inclusivity, and the implications of analytic choices. The session concluded with group presentations of the revised teaching materials, followed by a collective debrief and synthesis of emerging themes. Group 1 focused on regression modelling and missing data, while group 2 examined interaction/effect measure modification.

## Results

The following case examples summarize key insights from small-group discussions of selected textbook examples, highlighting how participants reinterpreted teaching materials through an EDI perspective.

### Case example 1A: regression modelling

Group 1 participants discussed Example 11.9 from *Fundamentals of Biostatistics* (p. 464), which presents a bivariate association between estriol levels and birth weight. The original example referred to the study sample as “pregnant women,” prompting reflection on how language shapes inclusivity. Group 1 recommended using “pregnant person” as the default phrasing unless the target population is explicitly defined as cisgender women (Statistics Canada, 2021). This distinction helps clarify scope while ensuring that teaching examples do not unintentionally exclude gender-diverse individuals.

Participants observed that the example’s use of the terms “normal” and “abnormal” estriol levels could imply value judgments without clarifying the clinical or population context. While recognizing that biological benchmarks are important for clinical interpretation, the group discussed how such thresholds are established within specific biomedical, social, and historical contexts and may not capture the full range of population variability. Group 1 emphasized that teaching examples should encourage students to consider what factors such as nutrition, stress, or structural inequities might contribute to observed differences in estriol levels, rather than attributing variation solely to individual biology. Group 1 also noted that the example implicitly assumed the pregnant person intended to continue the pregnancy, and they recommended that such assumptions be explicitly stated or contextualized to avoid reinforcing normative views of pregnancy. The revised framing highlighted how “normality” is defined within particular social, biological, and structural conditions, inviting reflection on how analytic choices intersect with broader determinants of health.

The discussion expanded to consider a broader range of confounding factors that could influence both estriol levels and birthweight, including malnutrition, ethnicity, maternal age, diabetes, viral infections, socioeconomic status, and cortisol levels. The revised example incorporated these factors to enhance teaching on how to specify and interpret regression models. Participants also recommended linking this example to later discussions on DAGs, illustrating how social and biological determinants can intersect in causal reasoning.

### Case example 1B: missing data and selection bias

In discussing Example 13.77 from *Fundamentals of Biostatistics* (pp. 753–754), which presents a study of ageing exploring how factors such as age, sex, physical performance, and self-rated health relate to mortality among older adults, workshop participants identified several issues in framing the ideal example. The example used the term “elderly” and coded sex as 1 for male and 2 for female, with individuals described as “willing” or “unwilling” to perform a test. The group found this language problematic and recommended replacing “elderly” with “older adults” to avoid ageist connotations. They also proposed revising gender coding to accommodate gender-diverse participants and rephrasing “unwilling” as “unable” to emphasize contextual rather than moral explanations for non-participation.

Building on this critique, participants discussed developing a more relevant and inclusive example using mental health survey data, such as the GAD-7 anxiety scale. They noted that nonresponse in such surveys is often systematic rather than random. For example, women or racialized individuals who experience higher anxiety may also be less likely to respond because of stigma, discrimination, or time constraints linked to structural inequities. Such patterns can create a missing-not-at-random mechanism, producing biased estimates and underestimating anxiety prevalence. The group reflected on how these biases are rarely addressed in textbook examples and how incorporating them can teach students to critically assess the assumptions underlying missing data mechanisms. Instructors might use this reworked example to demonstrate diagnostic tools for assessing nonresponse bias and to discuss how social determinants influence data quality and interpretation.

Participants used this reworked example to explore intersectionality (Lapalme et al., 2020), the overlapping social positions that shape health data patterns, and to illustrate the implications of bias for validity and inference. The discussion underscored the importance of framing statistical methods, such as imputation or weighting, within their social and ethical context. Participants emphasized that while these corrections are useful, they must be applied transparently and critically, recognizing that statistical adjustments cannot fully compensate for inequities embedded in data collection. This discussion reinforced the workshop’s broader goal of helping educators teach students to identify how methodological choices should be applied cautiously and transparently to avoid reinforcing structural inequities.

### Case example 2A: interaction/effect measure modification

Participants reviewed the section “Measures of Interaction and Scale of Interaction,” including Table 26–1 from *Modern Epidemiology* (pp. 619–620). The example presented an additive interaction between smoking and asbestos exposure in relation to the 10-year projected risk of lung cancer.

To build on this example, group 2 developed a parallel scenario using age as an effect modifier between asbestos exposure and the 10-year projected risk of lung cancer. In their adaptation, age was categorized as a binary variable, defining adults aged 65 and older as “older adults.” To clarify, the original example did not use binary age; rather, group 2 intentionally replaced the original *smoking (yes/no)* variable with binary age to illustrate effect modification by a social factor. This modification demonstrated how age, as both a social and biological construct, can modify the association between asbestos exposure and lung cancer risk, thereby grounding the statistical concept of effect modification in a more equity-relevant context. As a teaching example, this adaptation can help students consider how effect modification may reflect social and structural conditions rather than only biological differences between groups. For example, age may capture cumulative exposure to occupational hazards, housing conditions, environmental risks, or differential access to prevention and care.

Group 2 emphasized, however, that this approach must be applied thoughtfully. Age categories should be defined based on their conceptual relationship to the outcome of interest rather than an arbitrary cut-off. In this case, defining “older adults” as 70 years and older may be more appropriate, given that the average age at lung-cancer diagnosis is approximately 70 years. The group also noted that age can act as a proxy for multiple correlated risk factors, such as smoking history, exposure to second-hand smoke, or environmental hazards like radon in poorly ventilated housing. When used as a proxy, age becomes an amalgamation of several risk pathways. Teaching examples should therefore either (a) disaggregate these exposures using directly measured variables (e.g., smoking or second-hand smoke) or (b) explicitly acknowledge that age is serving as a proxy for multiple underlying risks.

Finally, participants used this case to briefly discuss age, period, and cohort effects. While these temporal dimensions are distinct from interaction or effect modification and may not necessarily be taught in conjunction, they are important contextual considerations when using age as an illustrative variable, as they highlight how generational and historical factors shape health outcomes (Harper, 2015).

### Case example 2B: hypothesis testing and two-way NOVA

Participants reviewed the section “Hypothesis Testing and Two-Way ANOVA” (pp. 591–594) from *Fundamentals of Biostatistics*. The example described an intervention in which participants were asked to consume two different diets: lacto-vegetarian and strict vegetarian. The study then examined the interaction between diet and sex, operationalized as male or female, in the association with systolic blood pressure. The group identified two main EDI considerations. The first concerned the biological basis for conducting the statistical test. Does the literature support the potential for an interaction between diet and sex on systolic blood pressure? The second consideration had to do with the measure of sex. As previously identified in examples from group 1, the definition of the sex variable is not provided and there is no discussion of gender.

Building on this critique, the group discussed potential considerations for the example, including adherence to diet and participation differing by sex. Discussion of the biological basis for the interaction test should be included in the teaching example. The teaching example could also incorporate a description of how sex was measured and a discussion of the limitations of this measure. If there is limited biological basis for the test, and the measure poorly defines biological sex, then the example may not be scientifically appropriate.

Group 2 proposed that a more relevant and inclusive framing might focus on food culture and ethnicity as modifiers of dietary intervention effects. These factors reflect meaningful differences in food preferences, cultural practices, and social determinants of health that can influence dietary adherence and health outcomes in intervention studies (Abrahamowicz et al., 2023; Bennett et al., 2022).

### Key findings from case examples

1. Use inclusive and precise language
  Terms such as “elderly,” “normal,” or binary labels for sex and gender may unintentionally marginalize or exclude groups. Reframing these terms (i.e., using “older adults” or “pregnant person”) may promote inclusivity and ensure teaching materials reflect diverse populations.
2. Define and measure constructs thoughtfully
  Variables like sex, gender, and age carry both biological and social dimensions. Educators should clarify what each represents, explain how it was measured, and discuss the implications of these choices for analysis and interpretation.
3. Frame statistical examples within social and biological contexts
  Teaching materials should invite critical reflection on whether interactions or associations have theoretical or empirical support. For example, before testing a diet–sex interaction, instructors can ask students to examine whether literature supports a biological basis or whether social factors (such as culture or access) are more relevant.
4. Recognize and address sources of bias
  Missing or nonresponse data, arbitrary cut-offs, and proxy variables often reflect structural and social inequities. Examples should demonstrate how these biases can influence inference and how transparent reporting or alternative modelling can mitigate inequitable conclusions.
5. Integrate intersectional and equity-focused reasoning
  Teaching should help students see how intersecting social positions (such as gender, race, and socioeconomic status) influence exposure and health outcomes. Embedding intersectional perspectives in statistical instruction supports critical thinking about data, determinants, and power.

## Discussion

This workshop reaffirmed that EDI integration is not an optional enhancement to epidemiology teaching but an essential element of methodological practice, rigor, and accountability. Participants recognized that inclusive education requires critical reflection on both what is taught and how it is taught. The process of revising textbook examples revealed how everyday teaching practices can perpetuate inequities through language, implicit assumptions, and the omission of marginalized perspectives. These reflections aligned with the workshop’s goals of helping educators identify bias in teaching examples and develop more inclusive ways to teach epidemiology and biostatistics.

Building on the foundations established in 2023, the 2025 session advanced the conversation from conceptual awareness to applied resource development. Participants not only identified problematic aspects of standard examples but also proposed actionable revisions. This shift from critique to creation marks a significant step toward embedding EDI in the routine practice of teaching epidemiology and biostatistics.

The discussion also highlighted the importance of institutional and structural alignment. Meaningful integration of EDI principles requires universities, public health agencies, and professional associations to support ongoing faculty development and provide accessible repositories of inclusive teaching materials. Additionally, participants underscored the value of positionality and reflexivity, encouraging instructors to acknowledge how their identities and experiences shape their pedagogical choices. By modelling reflexivity, educators can create more equitable and engaging learning environments. CSEB could support this work by offering recurring workshops or webinars on integrating EDI into epidemiology and biostatistics teaching, developing a shared repository of revised teaching examples and case studies, and creating facilitated working groups where educators and trainees can collaboratively refine course materials.

The next steps should focus on sustaining this momentum beyond a single conference workshop. Participants valued the opportunity to work through concrete teaching problems in small groups; subsequent conferences may continue this applied, discussion-based format rather than relying only on didactic presentations. Since topics were self-selected, the workshop placed greater emphasis on biostatistics than epidemiology; a more balanced structure may help ensure all learning outcomes are addressed consistently. These activities could help build a community of practice within CSEB and provide ongoing support for instructors seeking to integrate EDI principles into their teaching and practice. Finally, participants emphasized that integrating EDI in epidemiology and biostatistical education contributes to improved research quality. When students critically assess how variables such as race, gender, or socioeconomic status are conceptualized and measured, they gain a deeper understanding of bias, validity, and causal inference. Embedding EDI principles in teaching strengthens the ethical and methodological foundations of epidemiological and biostatistical inquiry. Continued collaboration between educators, students, and professional organizations will be key to ensuring that EDI principles remain embedded not only in classroom discussions but also in the broader discourse of the epidemiology and biostatistics fields.

## Implications for policy and practice

What are the innovations in this policy or program?

- This workshop translated broad principles of equity, diversity, and inclusion (EDI) into practical teaching strategies for epidemiology and biostatistics.
- By revising common textbook examples, participants identified how language, variable definitions, analytic assumptions, and framing choices can shape inclusion, representation, and methodological interpretation.
- This workshop offers a replicable, small-group model for developing inclusive teaching resources that can be adapted by educators, departments, and professional associations.

What are the burning research questions for this innovation?

- How can EDI principles be embedded within core epidemiology and biostatistics instruction rather than treated as separate from methodological training?
- What forms of faculty development, shared resources, and communities of practice best support inclusive teaching?
- How do inclusive teaching examples affect students’ understanding of measurement, bias, causal inference, structural determinants of health, and health equity?

## Data Availability

Not applicable.
